# Concomitant Antihyperalgesic and Antitumor Effects of Gabapentin in a Murine Cancer Pain Model

**DOI:** 10.3390/ijms22189671

**Published:** 2021-09-07

**Authors:** Beatriz Elena Brito, María Alejandra García, Yetsenia María De Gouveia, Pura Bolaños, Sindy Devis, Geraldinee Bernal, Víctor Alejandro Tortorici-Brito, Leslie Baute, Gabriel Díaz-Serrano, Víctor Tortorici

**Affiliations:** 1 Laboratorio de Patología Celular y Molecular, Centro de Medicina Experimental, Instituto Venezolano de Investigaciones Científicas (IVIC), Caracas 1020A, Venezuela; beatrizbrito2005@yahoo.com (B.E.B.); mariaalejandra_gm@yahoo.es (M.A.G.); yetsenia.mdgp@gmail.com (Y.M.D.G.); geraldineebernal@gmail.com (G.B.); tortorici97@gmail.com (V.A.T.-B.); lesliebaute1@yahoo.es (L.B.); 2 Laboratorio de Fisiología Celular, Centro de Biofísica y Bioquímica, Instituto Venezolano de Investigaciones Científicas (IVIC), Caracas 1020A, Venezuela; bolanosp@gmail.com; 3 Laboratorio de Neurofisiología, Centro de Biofísica y Bioquímica, Instituto Venezolano de Investigaciones Científicas (IVIC), Caracas 1020A, Venezuela; sindymdr@gmail.com (S.D.); gdiaz0890@gmail.com (G.D.-S.); 4 Laboratorio de Neurociencia, Departamento de Ciencias del Comportamiento, Escuela de Psicología, Universidad Metropolitana (UNIMET), Caracas 1073, Venezuela

**Keywords:** cancer pain, gabapentin, Cavα2δ subunit, tumor growth, hyperalgesia, allodynia

## Abstract

Cancer pain may be the consequence of physical nerve compression by a growing tumor. We employed a murine model to study whether gabapentin was able to regulate tumor growth, in addition to controlling hyperalgesic symptoms. A fluorescent melanoma cell line (B16–BL6/Zs green) was inoculated into the proximity of the sciatic nerve in male C57BL/6 mice. The tumor gradually compressed the nerve, causing hypersensitivity. Tumor growth was characterized via in vivo imaging techniques. Every other day, gabapentin (100 mg/Kg) or saline was IP administered to each animal. In the therapeutic protocol, gabapentin was administered once the tumor had induced increased nociception. In the preventive protocol, gabapentin was administered before the appearance of the positive signs. Additionally, in vitro experiments were performed to determine gabapentin’s effects on cell-line proliferation, the secretion of the chemokine CCL2, and calcium influx. In the therapeutically treated animals, baseline responses to noxious stimuli were recovered, and tumors were significantly reduced. Similarly, gabapentin reduced tumor growth during the preventive treatment, but a relapse was noticed when the administration stopped. Gabapentin also inhibited cell proliferation, the secretion of CCL2, and calcium influx. These results suggest that gabapentin might represent a multivalent strategy to control cancer-associated events in painful tumors.

## 1. Introduction

Cancer is often a painful disease that affects the quality of life. At least 15–20% of patients are likely to suffer from pain during the course of the disease, and an even higher proportion of the population becomes affected at its advanced stages [1,2]. Cancer-induced pain involves multiple symptoms such as hyperalgesia, allodynia, spontaneous pain, and numbness, which may be the consequence of physical nerve compression by the growing tumor, or direct infiltration of the nerve [2]; they may also result from tissue acidosis, the release of chemical algogens by the tumor, or the effects of cancer-prescribed therapies (surgery; chemo- or radiotherapy) [2].

Various cancer-induced pain models involving the inoculation of murine tumor cells into the hind paw or the thigh of mice have been developed [3,4,5,6]. Inoculation readily induced heat hyperalgesia and mechanical allodynia. These symptoms reached a maximum around two weeks later, at a time when signs of nerve compromise could be identified [3,4,5,6].

Since the proposal of the World Health Organization’s analgesic ladder [7], not only nonsteroidal anti-inflammatory drugs, but also weak or strong opioids have been employed to control cancer pain. Other drugs, such as metamizole (dipyrone), antidepressants, steroids, *N*-methyl-d-aspartate (NMDA) antagonists, and neuromodulators—including gabapentinoids—have also been used [2,3]. These drugs can be administered alone or in combination, and are usually indicated based on the intensity of pain, without considering—unfortunately—the mechanisms involved. On the other hand, cancer pain usually implies a prolonged use of analgesics and complications associated with their repeated administration [1,8,9]; thus, the search for a safer and more effective treatment remains a necessity.

Cancer-induced pain is mediated by the increased expression of voltage-gated calcium channels (VGCCs) [10]. Moreover, calcium signaling seems to be involved in cancer development processes, such as unregulated cell growth, resistance to apoptosis, enhanced angiogenesis, and invasion [11,12,13,14]. Overexpression of the alpha-2-delta subunit of VGCCs (Cavα2δ)—an auxiliary subunit that augments the ion conductance, promoted cell proliferation in vitro, and tumorigenesis in vivo—has also been noted [11,15,16]. Thus, regulation of VGCCs may represent an important mechanism to control both cancer pain and tumor progression.

On the other hand, gabapentin exerts an antihyperalgesic action through its binding to Cavα2δ of the L-, N- and P/Q-type VGCCs, modulating both presynaptic calcium influx and the subsequent calcium-mediated glutamate release [17], with the merit of causing only minor side effects at clinically effective doses. There are four Cavα2δ subtypes, and gabapentin binds to VGCCs’ alpha-2-delta-1 subunit (Cavα2δ1) with greater affinity compared to VGCCs’ alpha-2-delta-2 subunit (Cavα2δ2) [18,19,20,21,22,23,24].

Another molecule of particular importance in cancer development is the chemokine (C–C motif) ligand 2 (CCL2)—a key mediator of interactions between tumor and host cells, and also an essential participant in the development and maintenance of pain [10,25]. Treatment of dorsal root ganglion (DRG) neurons in vitro with CCL2 increased the amount of mRNA for Cavα2δ [10,26,27]. Cavα2δ, in turn, favors an increase in VGCCs’ density in the plasma membrane [21], as well as channel conductance. Evidence suggests that CCL2 is released from tumor cells and promotes phenotypic changes in sensory neurons, which might explain how tumor cells drive functional changes in nociceptors to cause hyperalgesia. Previous findings also indicate that injury to peripheral nerves triggers CCL2 release from afferents in the spinal cord’s dorsal horn, leading to pronociceptive effects and causing the production of proinflammatory factors [28]. Moreover, expression of CCR2—one of the CCL2 receptors—is increased in murine spinal cord neurons after peripheral nerve injury, and administration of CCR2 antagonists induces proanalgesic effects [29].

In this study, we employed gabapentin to evaluate its antihyperalgesic effect and also its possible antitumor action in a model of cancer-induced pain using male mice. Our findings suggest that in addition to controlling mechanical and thermal hypersensitivity, gabapentin also regulates tumor proliferation, CCL2 secretion, and calcium influx. These results indicate that using gabapentin to modulate pathophysiological calcium signaling in cancer might represent an effective multivalent therapeutic strategy.

## 2. Results

### 2.1. Tumor–Nerve Details

**Figure 1:**
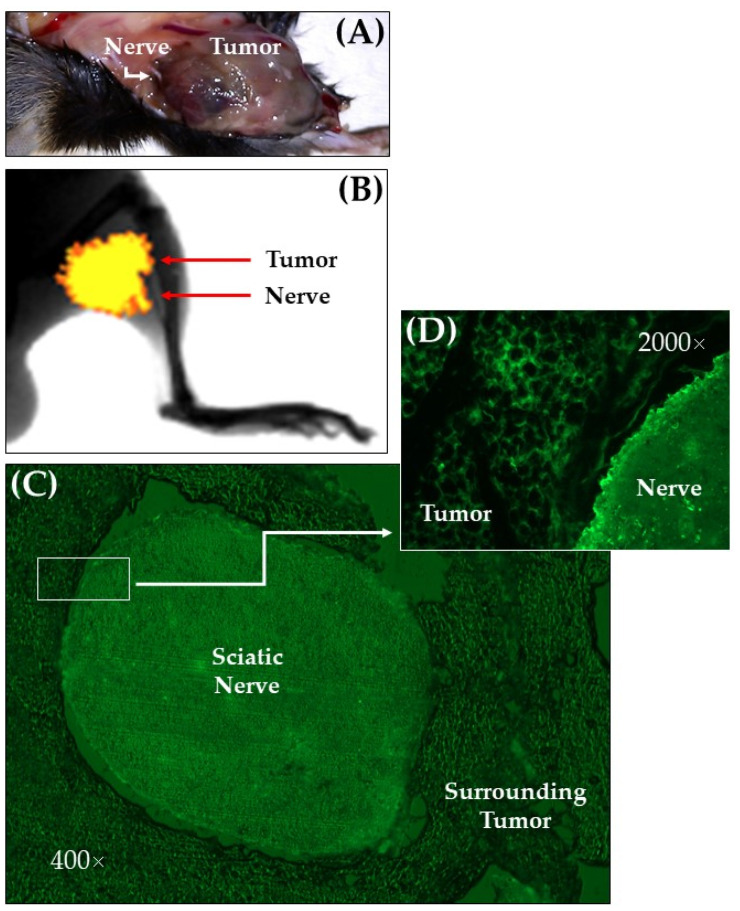
Tumor–nerve details: (**A**) Tumor mass engulfing the sciatic nerve. (**B**) In vivo image showing the tumor fluorescence signal co-registered with an anatomical X-ray plane of the inoculated hind paw. (**C**) Fluorescent microscope image (400×) of a transversal section of the sciatic nerve surrounded by the tumor. (**D**) Insert at higher magnification (2000×) showing the fluorescent B16–BL6/Zs green cells around the sciatic nerve. Images were taken 17 days after inoculation of the cell line (endpoint).

As depicted in Figure 1, after the inoculation of the tumor cell line, a fluorescent tumor grew in places limited to the outside of the epineurium, with the sciatic nerve surrounded by tumor tissue. The nerve was clearly distinguished from the surrounding tumor, without infiltration, degenerative changes, or motor dysfunction. Hence, the observed modifications in mechanical and thermal sensitivity of the hind paws (the sciatic nerve’s territory) could be the consequence of nerve injury, promoted by the progressive compression of a tumor that resembled a neurotropic melanoma or a melanotic schwannoma [3,30].

### 2.2. Nociceptive Changes Produced by Tumor Progression

**Figure 2:**
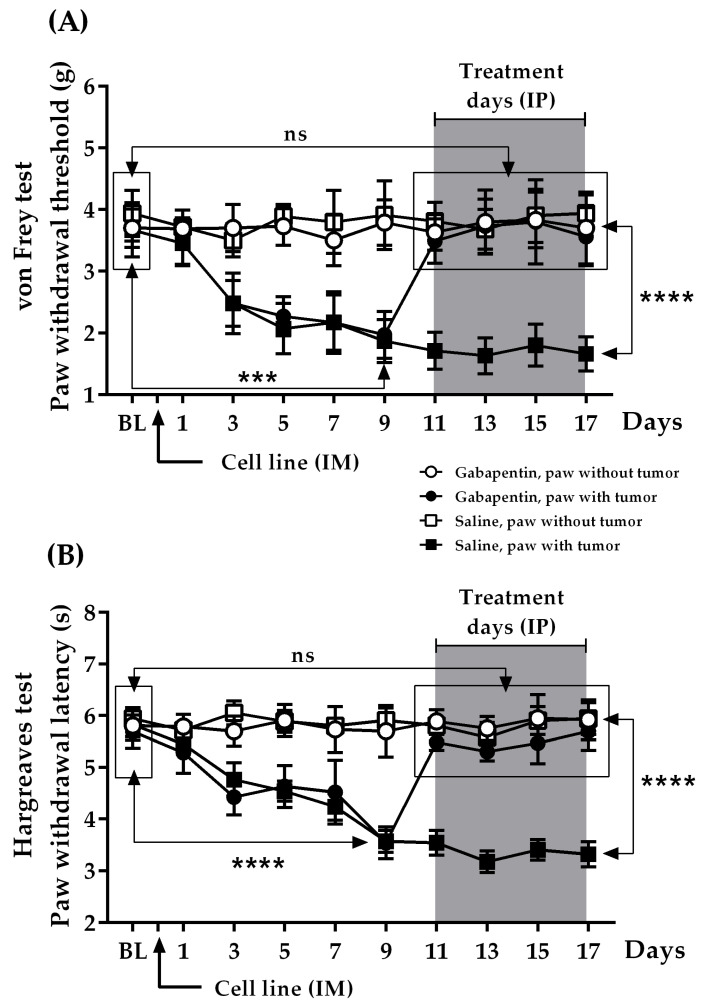
Therapeutic protocol: Gabapentin (100 mg/Kg) or saline was administered IP (days 11–17, grey box) to evaluate their effects on nociceptive responses caused by tumor progression: (**A**) von Frey test; (**B**) Hargreaves test. BL: Baseline (response value before IM inoculation of the cell line and before starting treatment). Contralateral non-inoculated limbs were used as internal controls. ns: non-significant. *** *p* ≤ 0.0002, **** *p* ≤ 0.0001, *n* = 10 mice/group. Each point represents the mean ± SEM of the sample.

As shown in Figure 2, the hind paws of the control animals had baseline (BL) values of 3.67 ± 0.18 g for the von Frey test (vF) and 5.83 ± 0.24 s for the Hargreaves test (HG). Once inoculated with the fluorescent melanoma cell line (B16–BL6/Zs green), and after receiving saline, nociceptive responses gradually increased from day 3, observing a decrease in the mechanical and thermal withdrawal thresholds that on day 9 reached 1.87 ± 0.35 g and 3.57 ± 0.21 s, respectively. These changes were only observed in the inoculated limbs (saline, paw with tumor day 9 vs. BL, *n* = 10, *p* ≤ 0.0002, 95% CI = −2.627 to −0.9731 for vF, and *p* ≤ 0.0001, 95% CI = −2.930 to −1.590 for HG). The response was stable from that moment, reaching 1.66 ± 0.28 g and 3.32 ± 0.24 s at the end of the experiment (saline, paw with tumor day 17 vs. BL, *n* = 10; *p* ≤ 0.0001 for both tests, 95% CI = −2.709 to −1.311 for vF, and 95% CI = −3.223 to −1.797 for HG).

### 2.3. Effect of Therapeutic Administration of Gabapentin upon Nociceptive Changes Produced by Tumor Progression

As soon as the therapeutic protocol was started on day 11 (Figure 2), gabapentin returned the behavioral responses of the inoculated limbs to BL levels in the experimental group (gabapentin, paw with tumor day 11 vs. BL, *n* = 10, 3.49 ± 0.36 g, *p* = 0.7552, 95% CI = −1.374 to 1.014 for vF, and 5.48 ± 0.15 s, *p* = 0.5598, 95% CI = −0.9525 to 0.5325 for HG). This effect was maintained throughout the experiment (gabapentin, paw with tumor day 17 vs. BL, *n* = 10, 3.56 ± 0.47 g, *p* = 0.8662, 95% CI = −1.463 to 1.243 for vF, and 5.70 ± 0.37 s, *p* = 0.9839, 95% CI = −1.018 to 1.038 for HG).

Based on these results, gabapentin administration promoted a significant antihypersensitive effect compared to the control group (gabapentin, paw with tumor vs. saline, paw with tumor, day 17, *n* = 10/group, *p* < 0.0001 for both tests, 3.65 ± 0.07 g vs. 1.70 ± 0.04 g, 95% CI = 1.746 to 2.144 for vF, and 5.49 ± 0.08 s vs. 3.36 ± 0.08 s, 95% CI for HG = 1.854 to 2.406 for HG). Nociceptive responses remained unchanged in control (non-inoculated) hind paws of the animals receiving the drug or saline (gabapentin or saline, paw without tumor, *n* = 10/group).

### 2.4. Effect of Preventive Administration of Gabapentin upon Nociceptive Changes Produced by Tumor Progression

**Figure 3:**
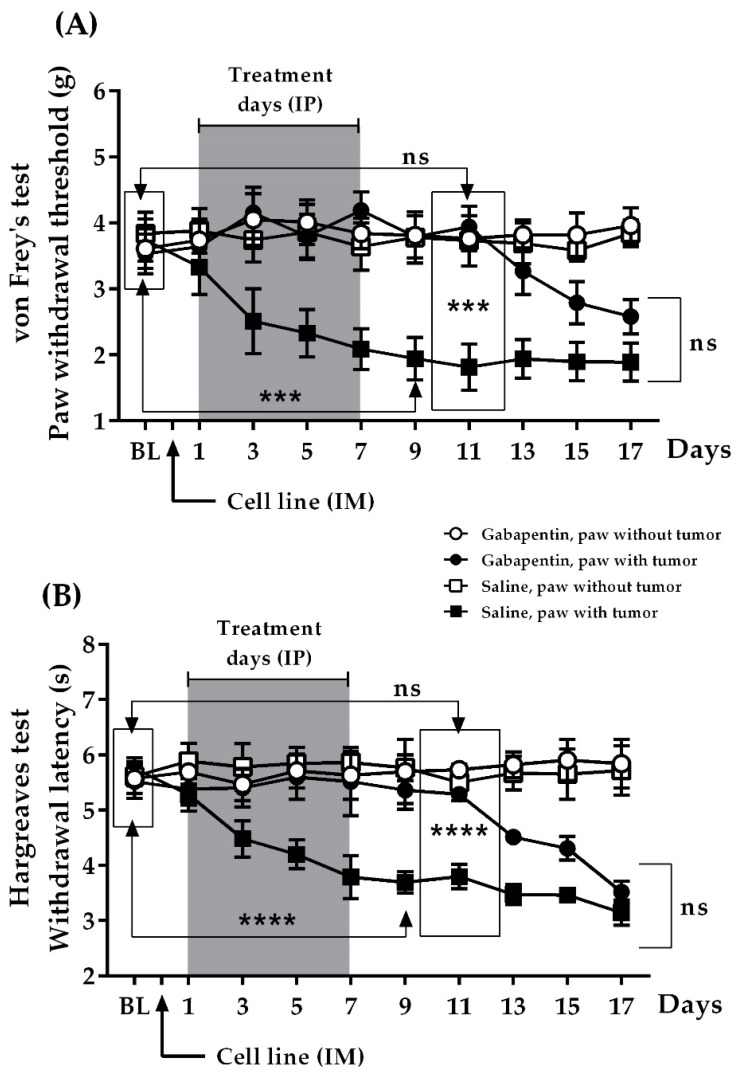
Preventive protocol: Gabapentin (100 mg/Kg) or saline was administered IP (days 1–7, grey box) to evaluate their effects on nociceptive responsiveness caused by tumor progression: (**A**) von Frey test; (**B**) Hargreaves test. BL: Baseline (response value before IM inoculation of the cell line and before starting treatment). Contralateral non-inoculated limbs were used as internal controls. ns: non-significant. *** *p* ≤ 0.0009, **** *p* < 0.0001, *n* = 10 mice/group. Each point represents the mean ± SEM of the sample.

As indicated in Figure 3, the hind paws of the control animals had BL values of 3.74 ± 0.32 g for vF and 5.75 ± 0.20 s for HG. In this group, there was, again, a progressive increase in nociception starting from day 3 post-inoculation, as observed with both tests, which tended to stabilize from day 9 (saline, paw with tumor day 9 vs. BL, *n* = 10; 1.94 ± 0.32 g, *p* = 0.0009, 95% CI = −2.748 to −0.8462 for vF, and 3.69 ± 0.19 s, *p* < 0.0001, 95% CI = −2.640 to −1.480 for HG) until the end of treatment (saline, paw with tumor day 17 vs. BL; *n* = 10, 1.89 ± 0.29 g, *p* = 0.0004, 95% CI for vF = −2.757 to −0.9427 for vF, and 3.14 ± 0.23 s, *p* < 0.0001, 95% CI = −3.250 to −1.970 for HG). However, the preventive administration of gabapentin maintained nociceptive responses at BL levels during treatment (3.53 ± 0.30 g and 5.52 ± 0.31 s, respectively). Interestingly, after ending administration, a delay of around 120 h was observed in the onset of the positive signs in the inoculated limbs (gabapentin, paw with tumor day 11 vs. BL, *n* = 10, 3.94 ± 0.31 g, *p* = 0.3545, 95% CI = −0.4963 to 1.316 for vF, and 5.29 ± 0.07 s, *p* = 0.4785, 95% CI = −0.8977 to 0.4377 for HG). This delay might be suggestive of either a residual effect of gabapentin or the existence of a critical time window for the onset of hypersensitivity. In fact, on that day, when comparing gabapentin-treated animals with their control inoculated counterparts, significant differences were observed (gabapentin, paw with tumor vs. saline, paw with tumor, day 11, *n* = 10/group, 3.94 ± 0.31 g vs. 1.81 ± 0.35 g, *p* = 0.0002, 95% CI = 1.144 to 3.109 for vF, and 5.29 ± 0.07 s vs. 3.80 ± 0.22 s, *p* < 0.0001, 95% CI = 1.005 to 1.975 for HG). From day 11, these limbs showed the characteristic increased nociception, so that by day 17, the responses were not statistically different to those of the control inoculated paws (gabapentin, paw with tumor vs. saline, paw with tumor, day 17, *n* = 10/group, 2.58 ± 0.26 g vs. 1.89 ± 0.29 g, *p* = 0.0934, 95% CI = −0.1283 to 1.508 for vF, and 3.52 ± 0.19 s vs. 3.14 ± 0.23 s, *p* = 0.2190, 95% CI = −0.2468 to 1.007). As expected for an antihyperalgesic agent, nociceptive responses remained unchanged in the non-inoculated paws of animals receiving either gabapentin or saline (gabapentin or saline, paw without tumor, *n* = 10/group).

### 2.5. Effects of Different Treatments on Tumor Growth

**Figure 4:**
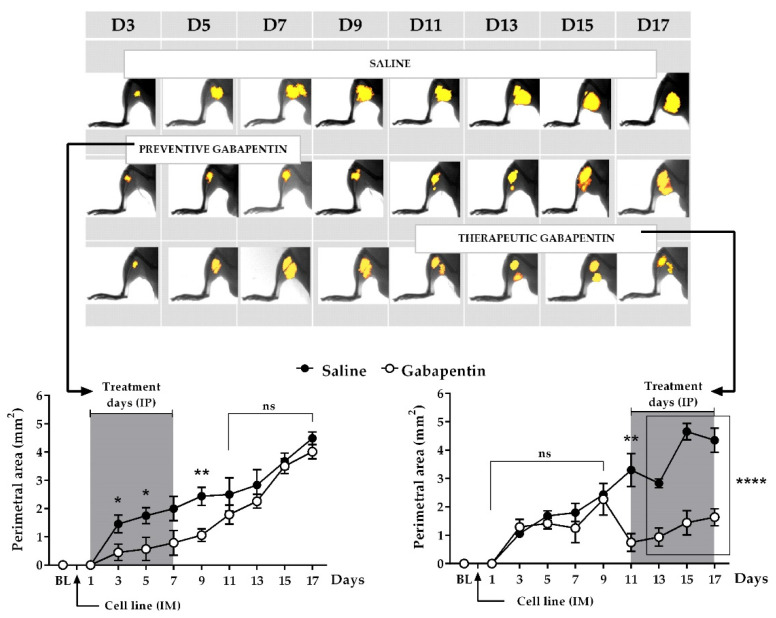
Tumor progression: **Above**: Representative image sequences, obtained with in vivo imaging techniques, 12 h after gabapentin administration, indicating tumor progression in three different intact mice receiving either IP saline (first row), preventive IP gabapentin (second row), or therapeutic IP gabapentin (third row) after the inoculation of the cell line. **Below**: Perimetral area (mm^2^), corresponding to all tumors growing in animals receiving either saline or gabapentin (100 mg/Kg). **Left**: Preventive treatment. **Right**: Therapeutic treatment. BL: Baseline (response value before IM inoculation of the cell line and before starting treatment). ns: non-significant. * *p* ≤ 0.0314, ** *p* ≤ 0.0024, **** *p* ≤ 0.0001, *n* = 10 mice/group. Each point represents the mean ± SEM of the sample.

A series of successive in vivo images, taken from the inoculated paws of three representative intact mice, 12 h after gabapentin administration, are shown in Figure 4 (above). The first row corresponds to an animal that received IP saline. As noted, the tumor was detectable from day 3 until the end of the experiment. Usually, the primary tumor was palpable from day 9 post-inoculation, so that the imaging techniques helped to explain behavioral changes even before the tumor was evident. The second row shows an example of how gabapentin prevented tumor growth; the effect was observed beyond the length of the treatment period, possibly indicating a residual effect of the drug or the existence of a critical time for the usual mass enlargement, which also matched the onset of hypersensitivity. However, after day 11, the tumor resumed its growth, reaching a size comparable to that in the control mouse (first row). The therapeutic effect of gabapentin is shown in the third row. As noted, the drug reduced tumor growth during treatment days and, in that mouse, the final size of the tumor was smaller compared to the other animals.

The perimetral area (mm^2^) of all tumors growing in mice that received either preventive (left) or therapeutic (right) treatment is shown in Figure 4 (below). During the gabapentin preventive treatment, with the exception of day 7, tumor growth was significantly reduced when compared with saline control animals (saline, paw with tumor vs. gabapentin, paw with tumor; day 3: 1.45 ± 0.32 mm^2^ vs. 0.45 ± 0.29 mm^2^, *p* = 0.0314, *n* = 10/group, 95% CI = −1.907 to −0.1004; day 5: 1.75 ± 0.28 mm^2^ vs. 0.57 ± 0.41 mm^2^; *p* = 0.0285, *n* = 10/group, 95% CI = −2.219 to −0.1392; day 7: 2.00 ± 0.43 mm^2^ vs. 0.79 ± 0.44 mm^2^, *p* = 0.0655, *n* = 10/group, 95% CI = −2.505 to 0.08635). This effect was observed until day 9 (2.44 ± 0.32 mm^2^ vs. 1.06 ± 0.22 mm^2^, *p* = 0.0024, *n* = 10/group, 95% CI = −2.194 to −0.5569), two days after finishing drug administration. From that point, the inhibitory effect of gabapentin on tumor growth gradually decreased, and tumors reached a similar size in both groups by the end of the assay.

When the animals (*n* = 10/group) received the therapeutic gabapentin treatment, tumor growth was significantly reduced (saline, paw with tumor vs. gabapentin, paw with tumor; day 11: 3.30 ± 0.59 mm^2^ vs. 0.75 ± 0.31 mm^2^, *p* = 0.0012, 95% CI = −3.943 to −1.156; day 13: 2.83 ± 0.15 mm^2^ vs. 0.94 ± 0.32 mm^2^, *p* < 0.0001, 95% CI = −2.641 to −1.147; day 15: 4.65 ± 0.29 mm^2^ vs. 1.44 ± 0.43 mm^2^, *p* < 0.0001, 95% CI = −4.299 to −2.119; day 17: 4.35 ± 0.42 vs. 1.64 ± 0.30 mm^2^, *p* < 0.0001, 95% CI = −3.800 to −1.621). Surprisingly, this effect was observed 12 h after the first therapeutic administration, and remained for the duration of the administration period, rendering a smaller tumor size by the end of the experiment.

### 2.6. In Vitro Proliferation Assays

**Figure 5:**
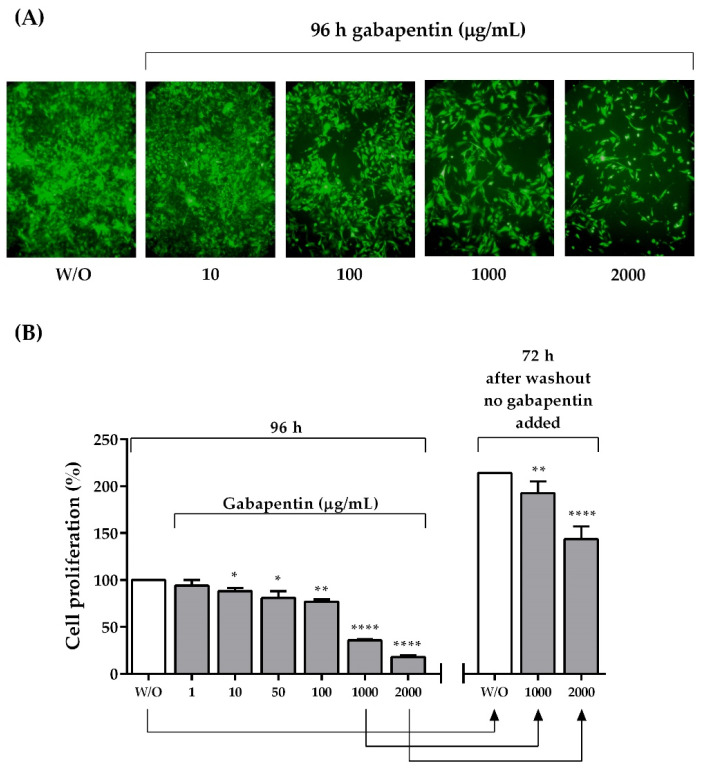
Cell proliferation: (**A**) Representative inverted fluorescent microscope images (200×) of the melanoma cell line B16–BL6/Zs green incubated for 96 h under different gabapentin doses (10, 100, 1000, or 2000 µg/mL). (**B**) Proliferation assay using MTT to verify viability. The results are expressed as a percentage of cellular proliferation after 96 h of incubation with different doses of gabapentin (1, 10, 50, 100, 1000, or 2000 µg/mL) compared with non-treated cells (100% proliferation), and after 72 h of gabapentin washout (no additional gabapentin added). Each column represents the mean ± SEM of the sample. * *p* ≤ 0.05, ** *p* = 0.0046, **** *p* < 0.0001.

Our results indicate (Figure 5A) that gabapentin was able to significantly reduce the proliferation of the melanoma cell line B16–BL6/Zs green in a dose-dependent manner. After 96 h of incubation with the drug, and compared to culture media without gabapentin (Figure 5B), the most inhibitory effective doses were 1000 μg/mL (35.78 ± 0.61% of proliferation, *n* = 6, *p* < 0.0001, 95% CI = −65.58 to −62.86) and 2000 μg/mL (18.12 ± 0.72% of proliferation; *n* = 6; *p* < 0.0001, 95% CI = −83.48 to −80.28). These results, obtained from MTT assays, suggest that instead of cytotoxicity, gabapentin seems to promote a cytostatic effect. Under these conditions, cell adhesion to the culture plate was maintained, and no dead cells were observed under the microscope. Interestingly, a significant relapse of cell proliferation was observed in the culture wells 72 h after washing gabapentin out of the plates, even for those that previously received the highest inhibitory doses (192.40 ± 5.54%, *n* = 5; *p* = 0.0046, 95% CI = −34.38 to −8.818 for the cells that were under 1000 μg/mL, and 143.00 ± 6.14%, *n* = 5; *p* < 0.0001, 95% CI = −85.15 to −56.85 for the cells that were under 2000 μg/mL). These findings support our previous in vivo observations using both protocols of gabapentin administration, and also during treatment suspension.

### 2.7. Chemokine CCL2 Determination

**Figure 6:**
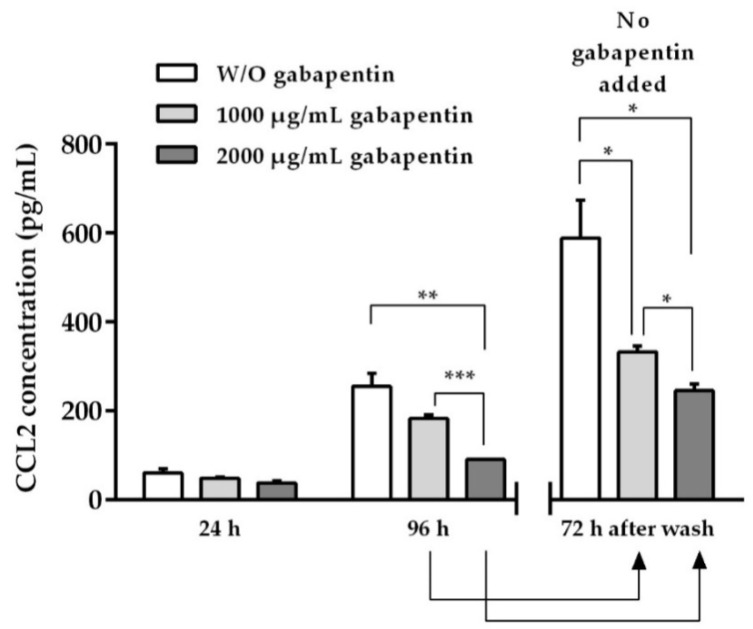
CCL2 concentration: Effect of gabapentin on the CCL2 secretion in B16–BL6/Zs green murine melanoma cells. Cultures were incubated with gabapentin (1000 or 2000 μg/mL) for 24 and 96 h, and 72 h after gabapentin washout (no additional gabapentin added). ELISA analyzed supernatants obtained from 3 independent assays (*n* = 3, each by triplicate), which were used to determine the chemokine concentration. Each column represents the mean ± SEM. * *p* ≤ 0.04, ** *p* ≤ 0.005, *** *p* ≤ 0.0005.

In supernatants obtained from our previous proliferation assays, CCL2 concentration was determined (Figure 6). Our findings indicate that B16–BL6/Zs green murine melanoma cells spontaneously secrete the chemokine CCL2 into the culture media (supernatants from cultures without gabapentin), contrary to that reported for B16-F10—another variant cell line of B16 murine melanoma that does not release CCL2 (59.44 ± 10.11 pg/mL at 24h, 255.00 ± 29.41 pg/mL at 96 h, and 588.03 ± 85.20 at 72 h after washout) [31,32]. When B16–BL6/Zs green cells were incubated during 96 h with the most effective doses of gabapentin (1000 or 2000 μg/mL), a dose-dependent inhibition of CCL2 secretion was observed (182.00 ± 9.05 pg/mL, *n* = 3, *p* = 0.0766, 95% CI = −158.4 to 12.43 for 1000 μg/mL, and 90.00 ± 0.66 pg/mL, *n* = 3, *p* ≤ 0.005, 95% CI = −246.7 to −83.32 for 2000 μg/mL vs. control supernatants without gabapentin). It should be noted that 72 h after removing gabapentin from the wells a significant residual effect of the previous incubation with the drug was still present (332.18 ± 13.68 pg/mL, *p* ≤ 0.04, *n* = 3, 95% CI = −495.4 to −16.27 for 1000 µg/mL, and 245.00 ± 14.52 pg/mL, *p* = 0.0166, *n* = 3, 95% CI = −583.0 to −103.0 for 2000 µg/mL vs. control without gabapentin), which resembled our in vivo observations.

### 2.8. Calcium Influx in B16–BL6 Melanoma Cells

**Figure 7:**
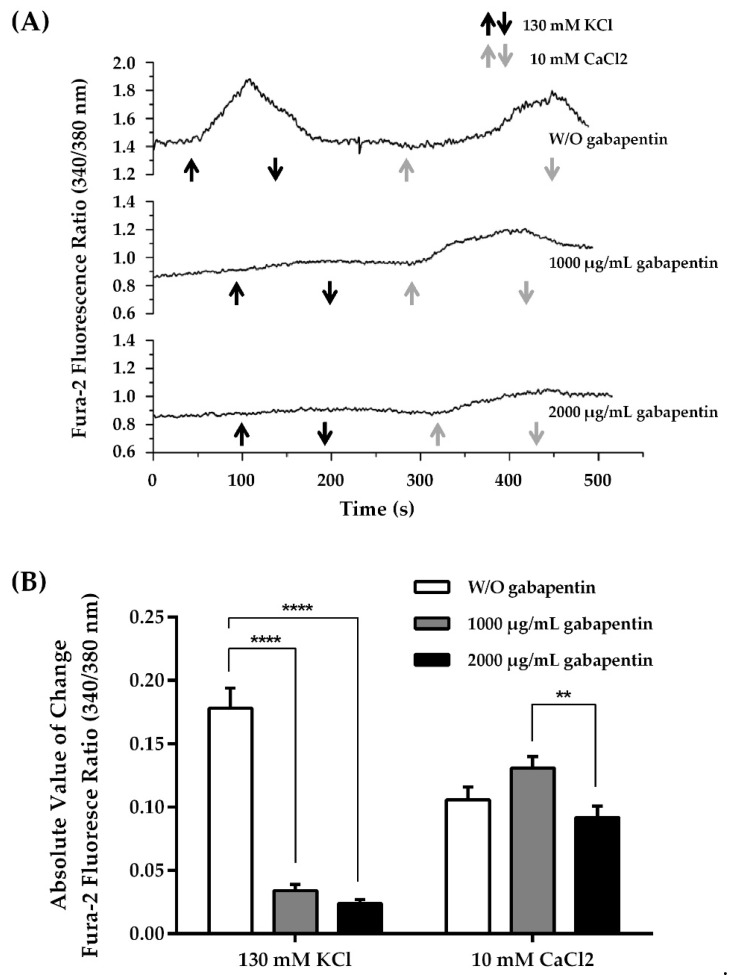
Calcium influx: Effect of gabapentin treatment on calcium influx in B16–BL6 murine melanoma cells: (**A**) Representative records showing changes in the time course of Fura-2 fluorescence ratio (340/380 nm) induced by 130 mM KCl or 10 mM CaCl_2_, in control and gabapentin-treated cells (1000 or 2000 µg/mL). Cells were preincubated with gabapentin for 24 h before starting the experimental procedure, and maintained throughout the recordings. When exposing the cells to 130 mM KCl (black ↑↓), gabapentin caused calcium influx inhibition. Once the basal calcium level was recovered in the control condition (1mM CaCl_2_), the subsequent exposure to 10 mM CaCl_2_ (grey ↑↓) induced an increase in calcium levels in all conditions. (**B**) Summarized data showing the absolute value of change (experimental condition baseline) of transient calcium responses after 130 mM KCl or 10 mM CaCl_2_ exposure in control and gabapentin-treated cells, expressed as Fura-2 fluorescence ratio (340/380 nm). Bars represent the mean ± SEM of data from control cells (*n* = 32) and gabapentin-treated cells (*n* = 17 under 1000 µg/mL, and *n* = 28 under 2000 µg/mL). ** *p* = 0.0061; **** *p* < 0.0001 vs. control.

Calcium influx was determined in murine B16–BL6 melanoma cells incubated for 24 h with gabapentin (1000 or 2000 µg/mL). Then, cells were loaded with Fura-2/AM, the high-affinity calcium-selective fluorescent indicator. Representative records from single melanoma cells showing the time course of the fluorescence ratio signal (340/380 nm) are shown in Figure 7A. After the initial observation of the baseline calcium influx (using a standard solution containing 1 mM CaCl_2_), a depolarizing condition was promoted by adding 130 mM KCl to the perfusion chamber. This mediated depolarization induced barely detectable changes in the cytosolic calcium levels in melanoma cells incubated with both doses of gabapentin (even though it was slightly higher in cells incubated with 1000 µg/mL). According to these results, gabapentin, through its binding to Cavα2δ of VGCCs, could modulate calcium influx in melanoma cells. However, in the control cell without gabapentin, high KCl-mediated depolarization triggered a marked increase in the fluorescence ratio. Later, after recovering baseline levels, the addition of calcium to the bath (10 mM CaCl_2_) caused a calcium influx response in all conditions, suggesting that cells treated with gabapentin were metabolically active, with the dose of 2000 µg/mL being the one that caused the greatest inhibition of calcium entry. Since gabapentin should affect the entry of ionized calcium through most VGCCs, this influx must involve different mechanisms.

As indicated from these representative traces, the starting point of the baseline in all of the gabapentin-treated cells showed lower fluorescence ratios (0.898 ± 0.012 for 1000 µg/mL, and 0.868 ± 0.009 for 2000 µg/mL, *p* = 0.0753) than the control cells (0.944 ± 0.014), probably reflecting the inhibitory effect caused by 24 h of preincubation with the drug (*p* = 0.0395, *n* = 17, 95% CI = −0.08857 to −0.003434 for gabapentin 1000 µg/mL, and *p* < 0.0001, *n* = 28, 95% CI = −0.1134 to −0.04461 for gabapentin 2000 µg/mL vs. control, respectively).

The summarized data showing the absolute value of change (experimental condition baseline) of transient calcium responses are shown in Figure 7B. After exposing the cells incubated with gabapentin with 130 mM KCl, calcium transients were significantly reduced (*p* < 0.0001 for both doses) compared to cells under the control condition (without gabapentin), which showed a marked increase in calcium influx (ratio 340/380 nm: 0.034 ± 0.005, *n* = 17 cells, 95% CI = −0.1890 to −0.09903 for gabapentin 1000 µg/mL; and ratio 340/380 nm: 0.024 ± 0.003, *n* = 28 cells, 95% CI = −0.1887 to −0.1193 for gabapentin 2000 µg/mL vs. control, ratio 340/380 nm: 0.178 ± 0.016, *n* = 32 cells). After returning to baseline conditions, exposure to 10 mM CaCl_2_ induced a calcium influx that was not significantly different from control cells (ratio 340/380 nm: 0.131 ± 0.009, *p* = 0.1077, *n* = 17 cells, 95% CI = −0.005664 to 0.05566 for gabapentin 1000 µg/mL; and 0.092 ± 0.009, *p* = 0.3079, *n* = 28 cells, 95% CI = −0.04124 to 0.01324 for gabapentin 2000 µg/mL vs. control, ratio 340/380 nm: 0.106 ± 0.010, *n* = 32 cells). These results indicate the persistence of calcium homeostatic mechanisms, suggesting that cells were still functional even after treatment with gabapentin.

## 3. Discussion

According to our results, gabapentin administration promoted an antihyperalgesic effect and tumor growth inhibition in a cancer-induced pain model employing male mice. These effects were observed in our in vivo and in vitro experiments, suggesting that in addition to controlling mechanical and thermal hypersensitivity caused by tumor engulfing of the sciatic nerve, gabapentin also regulates tumor proliferation, CCL2 secretion, and calcium influx. These results indicate that modulation of disrupted calcium entrance may be a potential target for cancer pain therapies.

Calcium influx can be considered a common denominator for the in vivo and in vitro effects observed herein. Ionic calcium is considered a universal second messenger and an essential regulator of the mitotic signal cascade, the cell cycle [12,13,14], and the cell’s proliferation mechanisms [33]. Likewise, calcium entry is also a key mediator of hypersensitivity in neurons of the pain pathway. The ultimate source of calcium is the extracellular microenvironment [11], and for that reason, its flow into the cells through specific membrane channels is fundamental for a correct cell function.

Calcium-dependent signaling is frequently deregulated in cancer cells and primary afferents compromised by a lesion (here for a surrounding tumor). In those scenarios, VGCCs play a crucial role in remodeling ion homeostasis. Hence, neuromodulators such as gabapentin—which regulates calcium entry through most VGCCs—may become a drug of choice to control hyperalgesia and tumor proliferation simultaneously. Available evidence indicates that calcium signaling plays a key role in melanoma viability [11,34,35,36], and our results are also consistent with this observation.

In the present study, we used a murine model of cancer-induced pain; it involved the inoculation of a fluorescent melanoma cell line in the vicinity of the sciatic nerve. Due to its fluorescence properties and the use of in vivo imaging techniques, we monitored tumor growth through the skin before it could be detected by thigh palpation, and studied early behavioral changes in advance. The tumor grew in place, limited to the outside of the epineurium, without infiltrating the nerve (at least during our time course). Our paradigm has been recently used [3], and resembled a rare, but not impossible, case of melanoma neurotropism [37,38] that causes tumor-related hyperalgesia in the inoculated hind paws (sciatic nerve’s territory). Additionally, we performed in vitro experiments to evaluate the effects of gabapentin on the proliferation of the melanoma cell line B16–BL6/Zs green, CCL2 secretion, and calcium influx.

Hyperalgesia is one of the most common and persistent symptoms associated with cancer, and multiple factors may contribute to the tumor-related increased nociception. Some of these may be attributable to the tumor itself, such as the release of peptides and other algogens, directly affecting sensory neurons [39,40]. Those neuroactive chemicals may also be released by immune cells in response to tumor-evoked damage to surrounding tissue, mechanical compression of nerves, or ischemia [41]. Several mechanisms underlying cancer-induced hyperalgesia may also involve components shared by neuropathic pain [42,43]; hence, the administration of agents commonly used to relieve pain-associated neuropathy may be similarly helpful in alleviating cancer-induced hyperalgesia.

Our behavioral results indicate that gabapentin significantly inhibits tumor growth and controls the increase in nociception. Before the onset of treatment, tumor-bearing animals showed emerging signs of mechanical allodynia and thermal hyperalgesia—even before the tumor was evident—which frequently occurred around day 9 post-inoculation. However, by day 3, imaging studies revealed small tumors already growing in the inoculated thighs. In saline control animals, hypersensitivity progressively increased until the end of the experiment and, by day 17, tumors reached the most extensive perimetral area. In the gabapentin group, as soon as the therapeutic protocol was started (day 11), the animals recovered their baseline level of response in the behavioral tests and, 12 h after drug administration, tumor mass significantly reduced its progression, appearing fragmented via imaging techniques. In mice receiving the preventive treatment, gabapentin induced a significant inhibition of tumor growth as soon as treatment started, and those animals did not show the typical initial increase in hyperalgesia. Nevertheless, after discontinuing treatment, tumor growth and hyperalgesia remained under control for four additional days, indicating the possible existence of a residual effect on cancer-induced nociception and cellular proliferation. These results suggest that cancer can and should be treated in advance to guarantee maximum control of the nociceptive symptoms and tumor growth.

Gabapentin—a derivative of the inhibitory neurotransmitter GABA—was initially designed as a GABAmimetic agent licensed to treat refractory epilepsy. It has gained wide recognition for its efficacy in controlling chronic pain syndromes, especially neuropathic pain [24], with minimal side effects. Even though gabapentin may induce dose-dependent motor incoordination and sedation at higher doses [44,45,46], in the present experiments, no sedation, catalepsy, or motor impairment was observed. The discovery of one specific binding site for gabapentin in the Cavα2δ subunit stimulated characterization of VGCCs in the drug’s clinical activity [2,19,24,43,47,48,49,50]. Current evidence favors the existence of four isoforms of Cavα2δ subunit, only two of which (Cavα2δ1 and Cavα2δ2) bind gabapentin [2] in L-, N- and P/Q-type calcium channels [24,49]. This binding promotes inhibition of calcium inward currents [51] and, consequently, leads to a reduced release of glutamate [24,52,53] and other excitatory molecules such as substance P [24,54] and NMDA [55], with the expected attenuation of postsynaptic excitability [2,19,24,47,56,57]. In this study, we observed a similar inhibition of calcium influx in murine melanoma (non-excitable) cells treated with gabapentin. Interestingly, while the Cavα2δ1 subunit has been involved with the effects of gabapentin against neuropathic pain, the Cavα2δ2 subunit seems to mediate some of the adverse effects of gabapentinoids, such as dizziness and sedation [58]. Hence, the chance of observing unwanted side effects should increase with higher doses, due to the increased probability of binding to the Cavα2δ2 subunit.

Furthermore, the reduction of glutamate release induced by gabapentin might modulate the occupancy of the metabotropic glutamate receptor 1 (GRM1), as well as the NMDA receptor, which seems to induce spontaneous melanoma development in vivo [59,60,61] and melanoma growth [62]. On the other hand, glutamate, acting through NMDA receptors, has also been implicated in changes of the natural killer cell response [63], which is endogenously responsible for reducing tumor activity.

Nerve damage is associated with changes in the electrophysiological and neurochemical properties of injured primary afferent neurons that cause the expression of a gain-of-function phenotype, resulting in activation at lower depolarizing membrane potentials. For example, DRG neurons showed increased ipsilateral expression of the Cavα2δ1 subunit following partial sciatic nerve ligation [64]; this upregulation influences the processing of sensory information via calcium-dependent mechanisms such as neurotransmitter release and neuronal excitability. In those cases, gabapentin reverted to allodynic behavior and inhibited peripheral ectopic discharges from injured nerve sites [65]. Whether or not similar changes can be promoted by a tumor growing around a nerve is certainly worthy of consideration. Interestingly, in a murine model of cancer pain, the implantation of fibrosarcoma cells into and around the calcaneus bone caused tumor-evoked heat hyperalgesia and sensitization, with ongoing spontaneous C nociceptor activity and increased wide-dynamic-range (WDR) neuron discharge [40,41]. In that model, tumor-evoked sensitization was associated with increased mRNA levels for the Cavα2δ1 subunit, and pretreatment with gabapentin blocked mechanical hyperalgesia [10].

A possible element that may contribute to explain the correlation between sensitization and Cavα2δ1 subunit overexpression is CCL2. Chemokines have been implicated in developing and maintaining pain [25], and CCL2 immunoreactivity was found in animals bearing a tumor [10]. Several lines of evidence suggest that CCL2 is a mediator of persistent hyperalgesia. CCL2 depolarizes DRG neurons in several models of neuropathic pain [10]. Moreover, CCL2 and its CC chemokine ligand-receptor 2 (CCR2) became upregulated in neurons after the DRG’s chronic compression [66,67]. Additionally, the development of mechanical allodynia was blocked in neuropathic CCR2 knockout mice [68], and the treatment of DRG neurons in vitro with CCL2 obtained from fibrosarcoma culture supernatants increased the amount of mRNA for the Cavα2δ1 subunit. These findings suggest that CCL2 released from tumor cells might promote phenotypic changes in sensory neurons, including overexpression of accessory subunits in VGCCs that likely underlie the mechanical hyperalgesia in the fibrosarcoma cancer model [10], and perhaps contribute to explaining the mechanical allodynia and thermal hyperalgesia also observed in our animals. We found that B16–BL6/Zs green melanoma cells secrete CCL2 spontaneously; thus, the tumor mass could directly affect the excitability of nociceptive neurons in our animals, and might induce the upregulation of CCL2 receptors (CCR2 and CCR4), as has been previously suggested [69].

The expression of VGCCs has been reported in melanoma cells, which supports their roles in tumorigenesis and tumor progression [15]. Aberrant expression and abnormal activity of specific ion channels were associated with increased cancer aggressiveness [70,71]. Therefore, pharmacological regulation of channel activity might offer protection against several types of cancers [15]. Melanoma cells express channel isoforms belonging to the *Cav1* (which mainly encodes high-voltage-activated L-type channels) and *Cav2* (which encodes high-voltage-activated P⁄ Q-type, N-type, and R-type channels) gene families [11]. Hence, agents that modulate these calcium channels—such as gabapentin—may become a therapeutic option to prevent melanoma growth, migration, or invasion—and, simultaneously, tumor-related hyperalgesia.

The fact that CCL2 is released by a compressed nerve [66,67]—and, constitutively, by our melanoma cell line—may explain why 12 h after starting the gabapentin administration in the therapeutic protocol, a significant decrease in tumor size was observed. As mentioned before, CCL2 induces overexpression of the Cavα2δ1 subunit in the VGCCs of primary afferents [10] and melanoma cells [15], as well as overexpression of CCL2 receptors (CCR2 and CCR4) [69]. These elements should improve the gabapentin binding and, consequently, its related effects on the spatial and temporal organization of calcium signaling and tumor growth inhibition. The combined overexpression of these elements over the course of the experiments should augment tissue distribution of the binding sites for gabapentin, hence promoting the inhibition of calcium-dependent tumorigenic pathways, such as in cell proliferation.

In our in vitro experiments, we studied calcium entry under KCl-mediated depolarizing conditions. In B16–BL6 melanoma cells, gabapentin inhibited calcium influx compared to control cells (without gabapentin)—an effect probably caused by the binding of gabapentin to the Cavα2δ subunit of most VGCCs. However, calcium entry promoted by the addition of CaCl_2_ to the bath was not affected by gabapentin treatment, suggesting the participation of other calcium entry mechanisms present in non-excitable cells, such as TRP channels, T-type VGCCs (belonging to *Cav3*), and the store-operated calcium entry (SOCE) [33,72], among others. Hence, a potential combination of gabapentin with a pharmacological channel regulator (i.e., a SOCE inhibitor) is worthy of study to verify an improvement in controlling cell proliferation in this and other tumor models.

Cell proliferation and survival were also studied in our in vitro experiments. In B16–BL6/Zs green melanoma cells, gabapentin inhibited proliferation in a dose-dependent manner, inducing a cytostatic effect since viability was not affected, as shown by an evident proliferation relapse after washing gabapentin out of the wells (even though a residual effect of the previous incubation with gabapentin was still present). These in vitro results may contribute to explaining the effect of gabapentin on tumor growth observed in our in vivo experiments, and support the hypothesis that malignant cells exhibit a strong dependence on calcium influx for disease progression [73]. Such inhibition may result strategically when surgery, or radio- or chemotherapy, becomes necessary.

## 4. Materials and Methods

### 4.1. Animals

**Figure 8:**
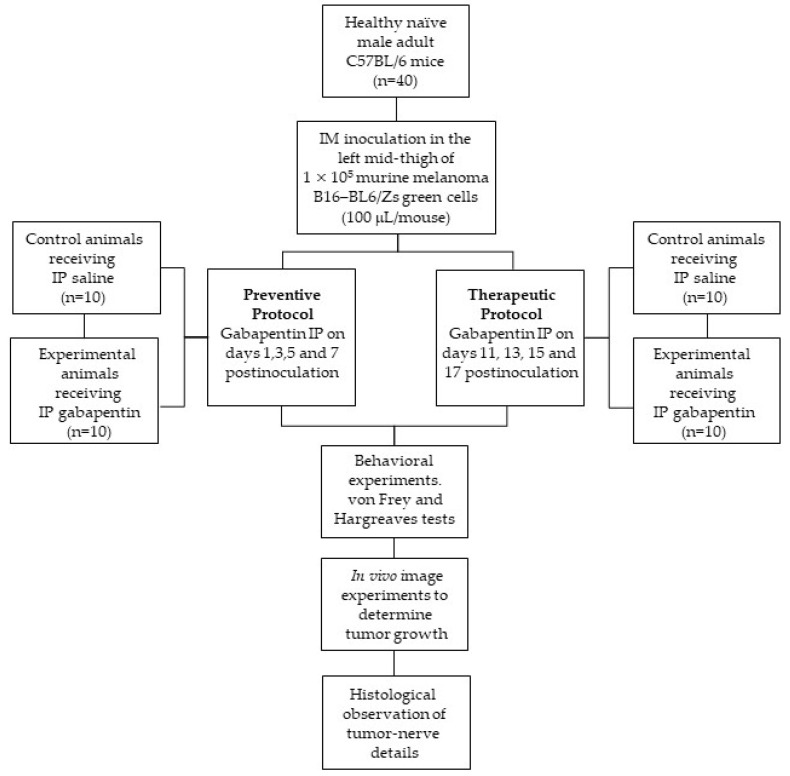
Summary of the in vivo experimental protocol.

Healthy naïve male adult C57BL/6 mice of 20–30 g (*n* = 10/group, for a total of 40 mice, including experimental and control animals) were used. This number was chosen based on power analysis (alpha = 0.1 and power = 0.9, suggesting around 9 animals for an expected analgesic effect of 1.0). To avoid possible hormonal effects, only male mice were included in the study. As indicated in Figure 8, for each experiment (therapeutic or preventive protocol), the study included two groups: (a) control animals: receiving IP saline; and (b) experimental animals: receiving IP gabapentin. Both hind paws were studied in each animal so that the contralateral non-inoculated limb represented an internal control. The animals were bred at the Central Animal Facility of the Instituto Venezolano de Investigaciones Científicas (IVIC).

Mice were kept in groups of five in plastic boxes with a microisolator filter on top. The colony room temperature was kept constant at 22 °C, with 25 air changes per hour. Humidity was also regulated, and light/dark cycles lasted 12 h each. All experiments were performed during the light cycle. Bedding for each cage and food (Ratarina, Alimentos Protinal, C.A., Valencia, Venezuela) were sterilized in a cobalt-60 plant at the IVIC. Water was delivered after a double-filtration process and, like the food, was available ad libitum. All experiments were performed in the Laboratorio de Neurofisiología, Centro de Biofísica y Bioquímica (CBB) at the IVIC, under the supervision (group allocation, experimental procedure, outcome assessment, and data analysis) of a responsible experimenter (V.T., corresponding author), and with the assistance of our veterinary staff to guarantee the health status of the animals. To minimize potential confounders (order of treatments, protocols, or animal location) a Google form was followed every experimental day. Fever, weight loss, decrease of water intake, sedation, motor impairment, catalepsy, spontaneous pain, or infection were continuously used as criteria established a priori to exclude animals during the study. However, no animals were excluded, and no unexpected adverse events were observed. At the end of each assay, mice were euthanized with an overdose of sodium thiopental (SM Pharma Laboratories, Caracas, Venezuela), following the recommendations of the Panel on Euthanasia of the American Veterinary Medical Association [74] and the Code of Practice for the Housing and Care of Animals Used in Scientific Procedures [75].

### 4.2. Drug Used

Gabapentin—1-(aminomethyl)-cyclohexaneacetic acid—was kindly donated by Pfizer, Inc., New York, NY, USA. A dose of 100 mg/Kg was chosen based on previous works [76,77,78] for intraperitoneal (IP) injection. The drug was in its pure state, which avoided additional experiments to monitor the potential effects of excipients. This was the lowest effective dose when used in our acute pilot experiments with C57BL/6 tumor-bearing mice (data not shown here for space reasons). Its maximum effect (at 30 min post-injection) was determined in previous acute experiments of 120-min duration; in those experiments, the behavioral tests (see below) were applied with an interval of 15 min between timepoints. During this observation period, neither sedation nor motor impairment was observed in the injected mice, similarly to a previous report from another group [76]. Gabapentin was dissolved in physiological solution (0.9% NaCl; Laboratorios Behrens, Caracas, Venezuela), and 0.2 mL was the injection volume. An equivalent volume of saline was administered IP to control animals.

### 4.3. Experimental Model

Our experimental model was employed here and previously [3] to quantify the nociceptive behavioral consequences of a tumor–nerve interaction, and its possible pharmacological management. As shown in Figure 8, this involved the use of murine melanoma B16–BL6/Zs green cells (kindly donated by Dr. Frank Marini, MD Anderson Cancer Center, Houston, TX, USA)—a transfected cell line that expresses the green fluorescent protein (GFP), derived from the highly invasive B16–BL6 melanoma line that has also been employed in a skin cancer pain model in C57BL/6 mice [5]. After light sedation, obtained by placing each animal in a ventilatory capsule containing halothane in oxygen, C57BL/6 mice were inoculated once, intramuscularly (IM), in the left mid-thigh (1 × 10^5^ cells/100 μL/animal), in the immediate vicinity of the sciatic nerve, as described in a neuropathic cancer pain model already published [6]. Halothane (Hoechst Marion Roussel, S.A., Caracas, Venezuela) was chosen since it provides ideal anesthesia for small animals, avoiding the risk of hypoxia and a rapid awakening time. The cell line was cultured, using conventional techniques, in low-glucose Dulbecco’s modified Eagle’s medium (DMEM) (Sigma-Aldrich Laboratories, Saint Louise, MO, USA) with 5% fetal calf serum (Invitrogen Corporation, Carlsbad, CA, USA), 1% penicillin–streptomycin (Sigma-Aldrich Laboratories), and 1% glutamine (Sigma-Aldrich Laboratories), maintained at 37 °C and 5% CO_2_ in a humid chamber.

### 4.4. Drug Administration Protocol

To evaluate the effects of gabapentin, each group of mice received a single IP injection of the drug or saline from a blinded experimenter, always at 09:00 a.m., every other day, for 4 days, to minimize the effect of excessive handling. Both experimental and control animals were randomly assigned. Each animal was identified with a number and allocated to its respective group using a random number generator (Decision Analyst STATS 2.0, www.decisionanalyst.com, accessed on 18 January 2019). The drug was administered on days 11, 13, 15, and 17 post-inoculation in the therapeutic protocol. These days corresponded to the maximum expression of tumor growth and significant changes in pain threshold [3]. The preventive treatment was carried out on days 1, 3, 5, and 7 post-inoculation, beginning before the primary tumor was palpable, and before the appearance of changes in pain threshold [3] (Figure 8). This latter protocol was intended to assess the possible existence of a “critical time window” for treatment, before the onset of pain signs and before the appearance of superficial morphological changes.

### 4.5. Behavioral Tests

On the experimental days, the von Frey and Hargreaves tests were applied to the plantar region of the hind paws (sciatic nerve’s territory), and withdrawal responses were observed before (baseline, BL) and after inoculating the cell line, as well as before and after drug or control vehicle administration. The von Frey test involves using a series of nylon monofilaments (Touch-Test Sensory Evaluator, Semmes–Weinstein monofilaments, Stoelting, Wood Dale, IL, USA) into the paw of each animal to apply a specific bending pressure depending on the calibrated filament employed. This pressure was expressed in grams (g). The animals were allowed to habituate in the chamber for a 5-min period. After that, the filaments were applied in ascending or descending sequence to determine the minimum pressure (threshold) that evoked a clear withdrawal response. This procedure was performed three times, with 3 min between tests, so that the results expressed in the graphs are an average of these measurements. The response of the contralateral limb (not inoculated) served as an internal control. The Hargreaves test involves placing each animal in a paw analgesiometer (Hargreaves, IITC Life Science, Series 8, Woodland Hills, CA, USA). The device has a transparent plastic chamber with a glass floor preheated to 28–30 °C. The animals remained in the chamber for a 5-min period of habituation. A thermal nociceptive stimulus (50 °C) was then applied, using an incandescent light source with an illumination area of 7 mm^2^. The time (latency) it took the animal to withdraw its paw from the radiant heat beam was determined. The cutoff time was 30 s. This procedure was applied three times, with a 5-min separation between trials, so that the results shown in the graphs represent an average of the three measures. Again, the unaffected contralateral paw served as an internal control.

### 4.6. Determination of Tumor Growth

Tumor growth was monitored in control and experimental animals using a Kodak FX In-Vivo Image Station (Carestream Inc., Woodbridge, CT, USA) (Figure 8). The equipment allowed the co-registration of the cell line’s fluorescence signal (ex465 and em535 nm filters for 30 s) with anatomical X-ray imaging (35 Kvp/30-s exposure, employing the same field of view and focal plane as the fluorescence image). All measurements were performed during the experimental days in lightly sedated animals (halothane vapors, see above) to minimize the stress associated with handling and capture a clear and stable image. Additionally, mice were placed onto a warmed stage inside the security cabinet for the duration of the exposure. Images were taken 12 h after gabapentin administration due to limited access to the equipment. Changes were detected only in the left mid-thigh of the live animals, even before superficial morphological changes could be detected via palpation. Images were obtained without euthanizing the animals for the whole experimental period, which reduced the number of animals in the study. Regions of interest (ROIs) from displayed images were identified and used to calculate the perimetral tumor area (mm^2^). At the end of the process, the fluorescence images were pseudocolored to improve the contrast using the Kodak Molecular Imaging v4.5 software that comes with the equipment.

### 4.7. Histology

At the end of the experiments (day 17), mice were euthanized in a CO_2_ chamber, and tumor-bearing thighs were removed from each animal, fixed in 10% formalin for at least 24 h, and maintained in 70% ethanol before decalcification with Osteomol (Merck, Darmstadt, Germany) for 72 h. Subsequently, tissue was processed and embedded in paraffin using an automatic processor (Leica Microsystems TP 1020, Nussloch, Germany). For analysis purposes, 5-micron paraffin sections were stained with hematoxylin and eosin (H–E; Sigma-Aldrich, St. Louis, MO, USA) in an automatic stainer (Leica Microsystems Autostainer XL). Fluorescence observations from different fields at 400x and 2000x magnification were carried out using an inverted fluorescence microscope (DMIL LED, Leica Microsystems, Wetzlar, Germany) coupled with a camera (DFC310 FX, Leica Microsystems). Images were processed using the Leica LAS AF software, version 4.4 (Leica Microsystems).

### 4.8. In Vitro Proliferation Assays

**Figure 9:**
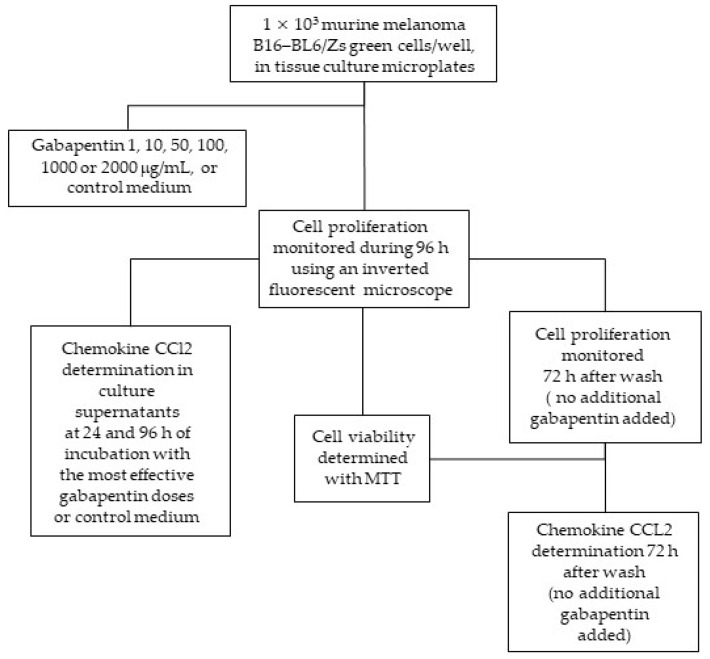
Summary of the in vitro experimental protocol.

The effect of gabapentin was also studied in vitro using conventional techniques, as mentioned previously. Murine B16–BL6/Zs green melanoma cells were seeded into 96-well flat-bottom tissue culture microplates (1 × 10^3^ cells/well, Falcon, Becton Dickinson, NJ, USA) and incubated for 24 h to allow adherence to the plate. Thereafter, six different concentrations of the drug (1, 10, 50, 100, 1000, or 2000 mg/mL) were tested (Figure 9); these concentrations were chosen based on previous works [79,80,81]. According to pharmacokinetic studies, after the administration of a 400-mg gabapentin tablet to a human, its maximum concentration in plasma reached approximately 20 µM [80]. In this study, the cytostatic effect of gabapentin was determined using 2000 µg/mL as the highest dose—which is equivalent to 11.68 µM—and compared to the control cell cultures without gabapentin. According to a recent study [81], this concentration is similar to a third of the maximum daily dose of gabapentin (3600 mg). Cell proliferation was monitored 96 h later using an inverted fluorescence microscope (DMIL LED, Leica Microsystems) coupled with a camera (DFC310 FX, Leica Microsystems). Images were processed using the Leica LAS AF software, version 4.4 (Leica Microsystems).

The antiproliferative effect of the drug was studied in another set of culture plates. After completing the incubation time, gabapentin was washed out twice with 100 μL/well of phosphate-buffered saline (PBS), and the wells were replenished with culture media. Again, cell proliferation was monitored 72 h later. MTT (3-(4-5-dimethylthiazol-2-yl)-2,5 diphenyltetrazolium bromide; Sigma-Aldrich Laboratories, MO, USA; 2.5 mg/mL, 25 mL/well) was added, and plates were incubated for 4 h to test the viability of the cells after the drug challenge. When a cell is alive, MTT should be incorporated, and formazan crystals must be metabolically produced via a reduction process so that the amount of crystals reflects the number of viable cells present in the culture well [82]. Crystals were dissolved with a solubilizing solution (100 μL/well) containing 20% SDS in a 1:2 solution of N-N-dimethyl formamide (Fisher Scientific Co., Pittsburgh, PA, USA) in distilled water. Plates were incubated overnight in the dark and quantified spectrophotometrically (570 nm) using an ELISA plate reader (EL-307C; BioTek Instruments, Winooski, VT, USA). The results were expressed as a percentage of cellular proliferation compared to non-treated cells (100% proliferation). Each well condition was replicated three times per experiment, so that the results shown in the graphs represent an average of three measures.

### 4.9. Chemokine CCL2 Determination

Culture supernatants obtained from the B16–BL6/Zs green proliferation assays (see above) at 24 and 96 h of incubation with 1000 or 2000 μg/mL of gabapentin (most effective doses) were studied (Figure 9). Moreover, we included supernatants from culture plates after gabapentin was washed out, which were maintained with fresh culture medium for an additional 72-h period, as well as control supernatants from plates without gabapentin. A commercially sandwich enzyme-linked immunosorbent assay kit (ELISA Max^TM^ Standard Sets, BioLegend, San Diego, CA, USA) was used to determine the chemokine CCL2 protein concentration (pg/mL) on enzyme-linked immunosorbent assay microplates (MaxiSorp, Nunc^TM^, Apogent, Portsmouth, NH, USA). CCL2 concentration was estimated based on an appropriate internal standard curve using a recombinant murine chemokine. The results represent the mean of three independent assays, conducted in triplicate and expressed as the mean ± SEM.

### 4.10. Calcium Influx Measurements

**Figure 10:**
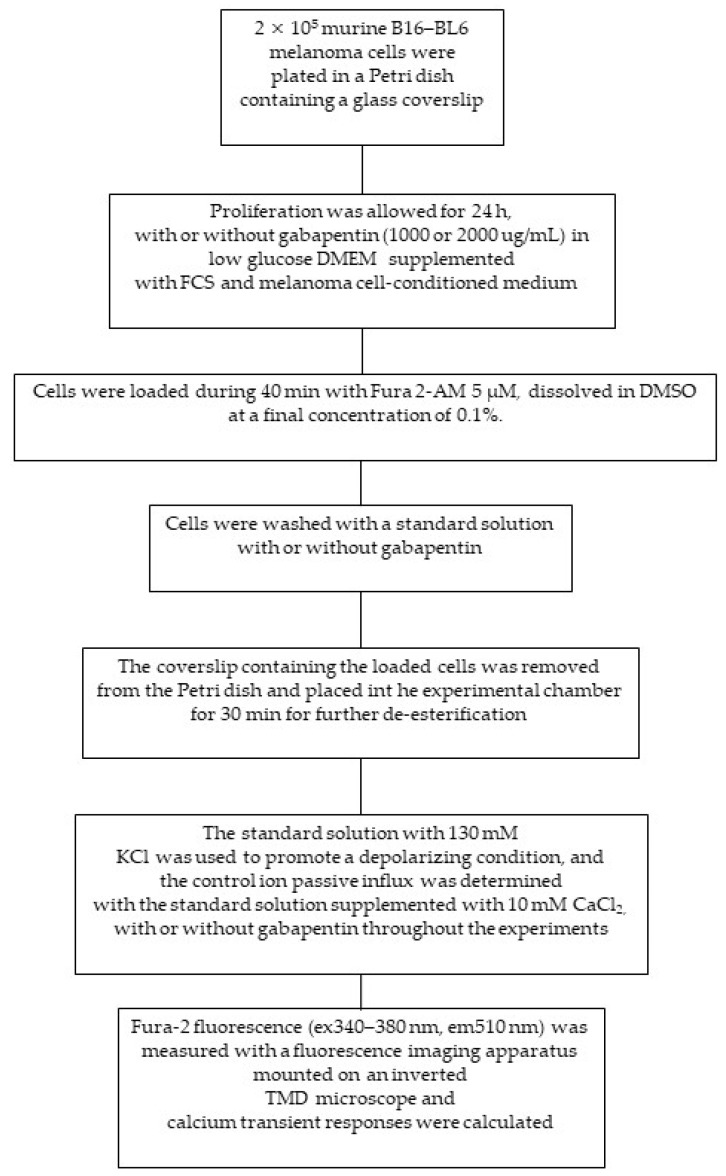
Summary of the in vitro calcium influx experiments.

Murine B16–BL6 melanoma cells (kindly donated by Dr. Peter Taylor, Centro de Medicina Experimental, IVIC, Caracas, Venezuela) were plated at a density of 2 × 10^5^ cells in a Petri dish (Sigma-Aldrich Laboratories) containing a glass coverslip (Corning Inc., New York, NY, USA) to determine the effect of gabapentin on calcium influx. Cells were allowed to proliferate for 24 h, with or without gabapentin (1000 or 2000 μg/mL), in low-glucose DMEM supplemented with 10% FCS, plus 40% melanoma-cell-conditioned medium, at 37 °C in a humidified atmosphere of 5% CO_2_ (Figure 10).

Later, B16–BL6 cells were loaded with 5 μM Fura-2/AM (Invitrogen, Molecular Probes, Eugene, OR, USA)—the high-affinity calcium-selective fluorescent intracellular indicator, dissolved in DMSO at a final concentration of 0.1% for 40 min, at 37 °C, in the dark. Then, cells were washed with standard solution (130 mM NaCl, 5 mM KCl, 1 mM CaCl_2_, 1 mM MgCl_2_, 10 mM HEPES, 10 mM glucose, pH 7.4; used as baseline condition), with or without gabapentin (1000 or 2000 µg/mL), throughout the recordings. The coverslip containing the loaded cells was then removed from the Petri dish and placed in an experimental chamber for 30 min to allow further de-esterification. The standard solution with 130 mM KCl was used to promote a depolarizing condition. The control ion passive influx was determined with the standard solution supplemented with 10 mM CaCl_2_.

Fura-2 fluorescence (ex340–380 nm, em510 nm) was measured with a fluorescence-imaging apparatus (IonOptix Co., Milton, MA, USA) mounted on an inverted Nikon Diaphot TMD microscope (Tokyo, Japan). For Fura-2 measurements, the light from a 100-W xenon lamp was filtered alternating 340/20- and 380/18-nm interference filters (Chroma Technology Corp., Rockingham, VT, USA). The resultant fluorescence was passed through a 400-nm dichroic mirror, filtered at 510/40 nm (Chroma Technology Corp., Rockingham, VT, USA), and collected using an intensified CCD camera. Fluorescence images were taken at a 33-ms/frame rate, digitalized, and analyzed using the IonOptix 5.0 software (IonOptix Co.). Results were graphed as the fluorescence ratio (340/380 nm) versus time (s) using Origin 8.1 software [83]. The absolute value of change of calcium transient responses was calculated by subtracting each experimental condition value from its baseline, and expressed as Fura-2 fluorescence ratio (340/380 nm). The murine melanoma cell line B16–BL6 was used in these experiments instead of the B16–BL6/Zs green cell line, since GFP interferes with the Fura-2 fluorescence signal. Previous work in our laboratory indicates that these cell lines are similarly affected by gabapentin in terms of in vivo tumor growth, as well as in vitro proliferation and CCL2 secretion.

### 4.11. Statistical Analysis

The results were reported as the mean ± SEM. The Kolmogorov–Smirnov test and Levene’s median test were performed to verify data normality. Then, differences between groups were evaluated by applying the Mann–Whitney *U*-test or the Kruskal–Wallis test. Differences were acknowledged as statistically significant when *p* ≤ 0.05. Confidence intervals were also included throughout the results section. Due to the absence of any exclusions, all of the animals were included in the analysis. The statistical program used was GraphPad Prism version 5.0 (GraphPad Software, San Diego, CA, USA).

## 5. Conclusions

Based on these results, the modulation of pathophysiological calcium signaling might represent an appropriate therapeutic strategy to control tumor development and progression, as well as tumor-induced hypersensitivity. The expected functional consequences of channel regulation must act not only on tumor parameters such as growth, migration, or invasion, but also on their sensorial and behavioral implications. However, careful selection of the ion influx modulator must be well thought out, because calcium channels are ubiquitously expressed in different tissues, playing relevant roles in normal cell function. Here, we present the alternative of gabapentin which, acting on Cavα2δ-bearing VGCCs of melanoma cells and primary afferents, seems to exert a concomitant antihyperalgesic and antitumor effect without apparent adverse effects, suggesting that this drug should be considered not merely as a coadjuvant, but also as a pharmacological regulator of calcium remodeling in pathophysiological conditions.

## Data Availability

All study data are available upon reasonable request after ethics clearance and approval by all coauthors for well-defined questions aligned with the overall research objectives.
